# Supplementary figures and images for: Mutagenic mechanisms of cancer-associated DNA polymerase ϵ alleles
Source: Nucleic Acids Res. 2021 Mar 25;49(7):3919–31. doi: 10.1093/nar/gkab160 (PMC8053093; doi:10.1093/nar/gkab160)

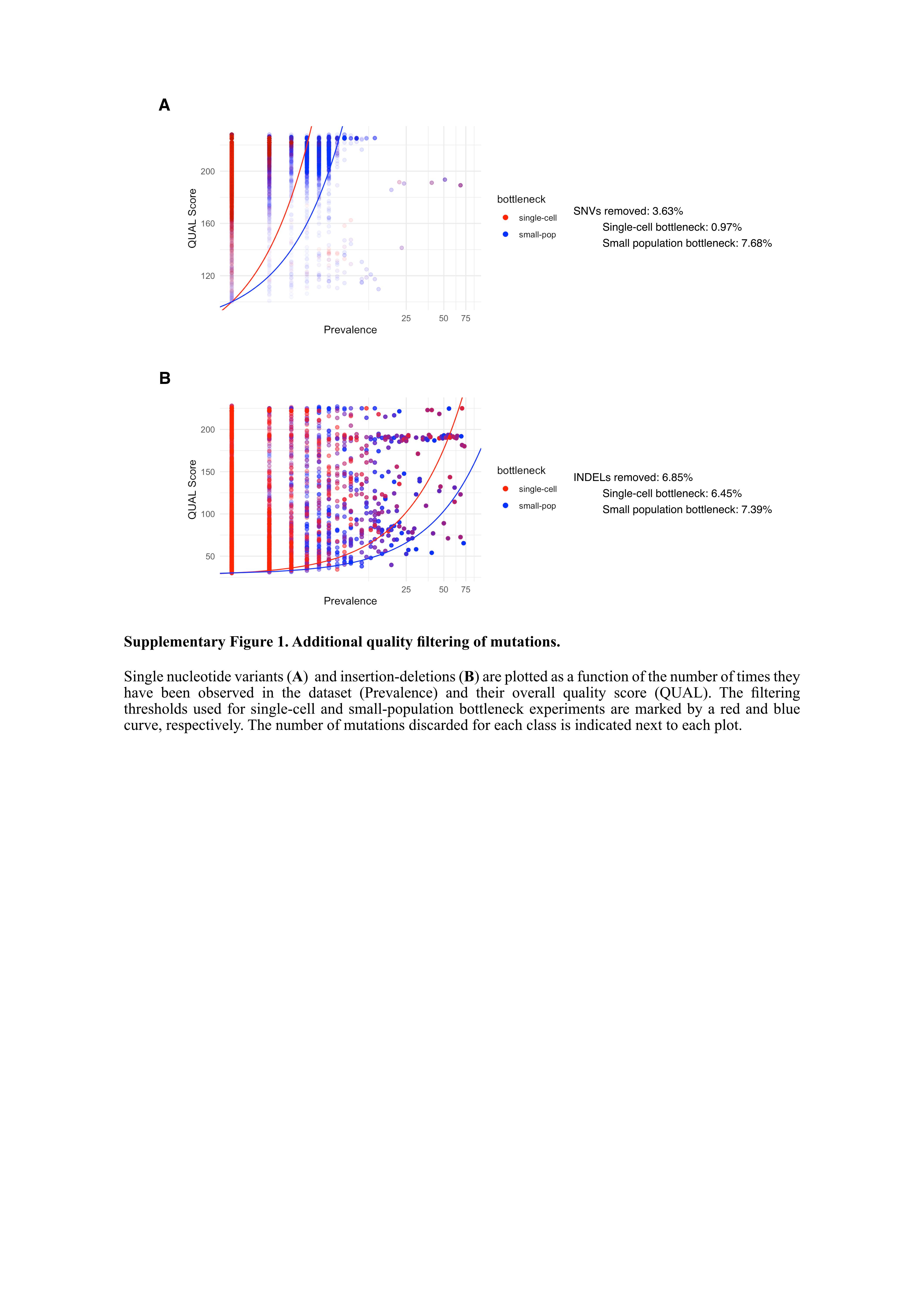

Supplement: gkab160_Supplemental_Files [file gkab160_supplemental_files.zip › SuppFig1.png]

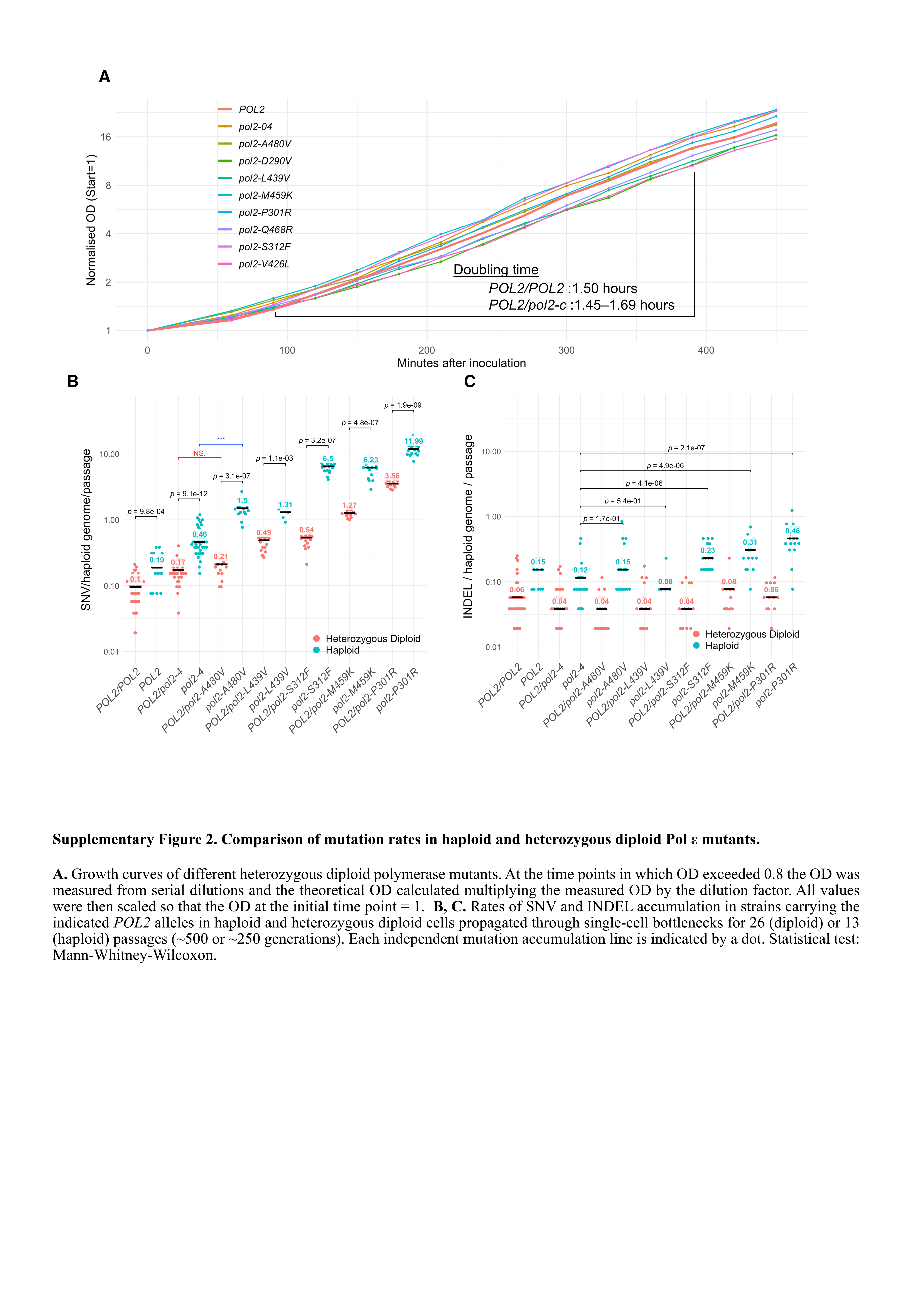

Supplement: gkab160_Supplemental_Files [file gkab160_supplemental_files.zip › SuppFig2.png]

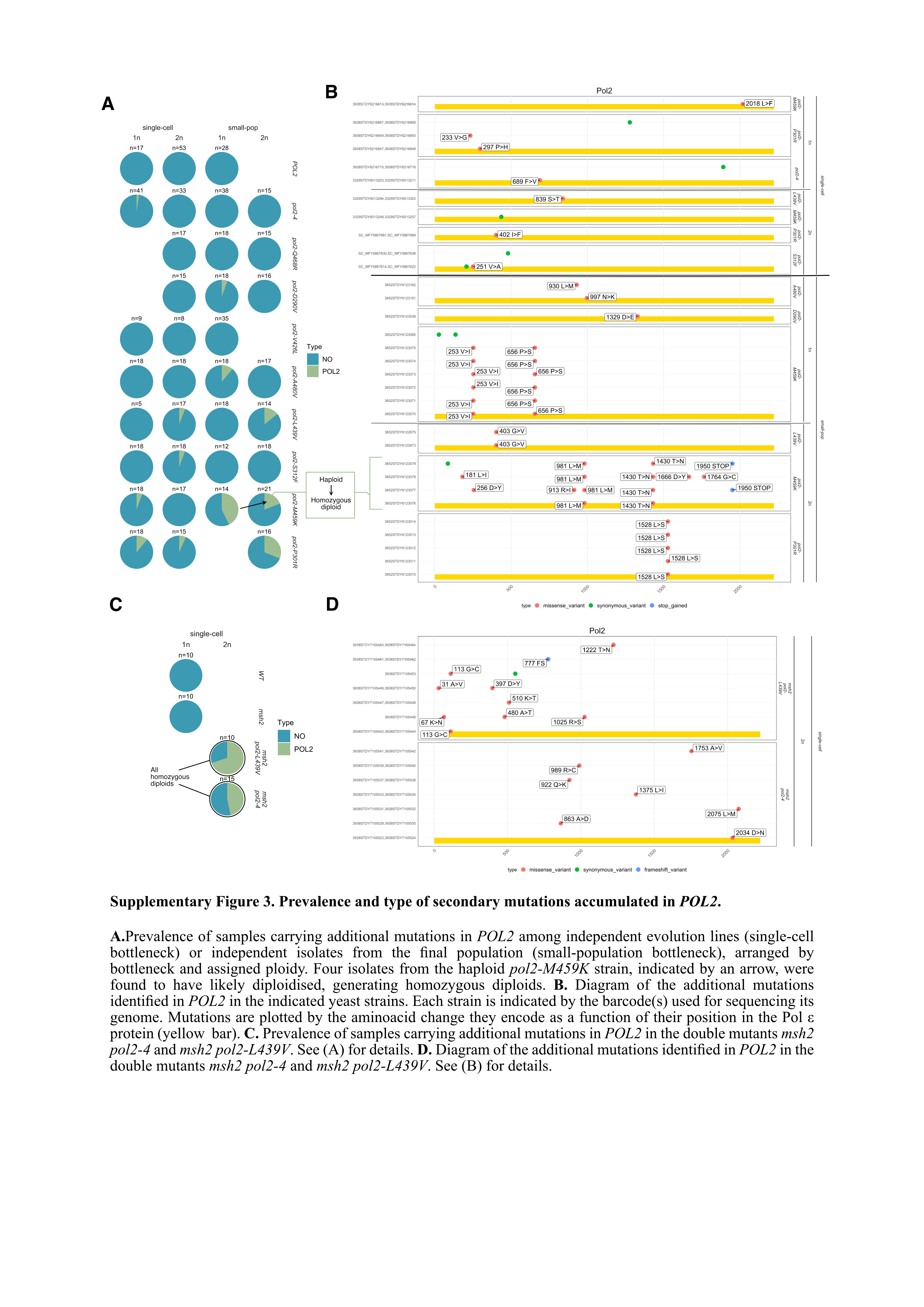

Supplement: gkab160_Supplemental_Files [file gkab160_supplemental_files.zip › SuppFig3.png]

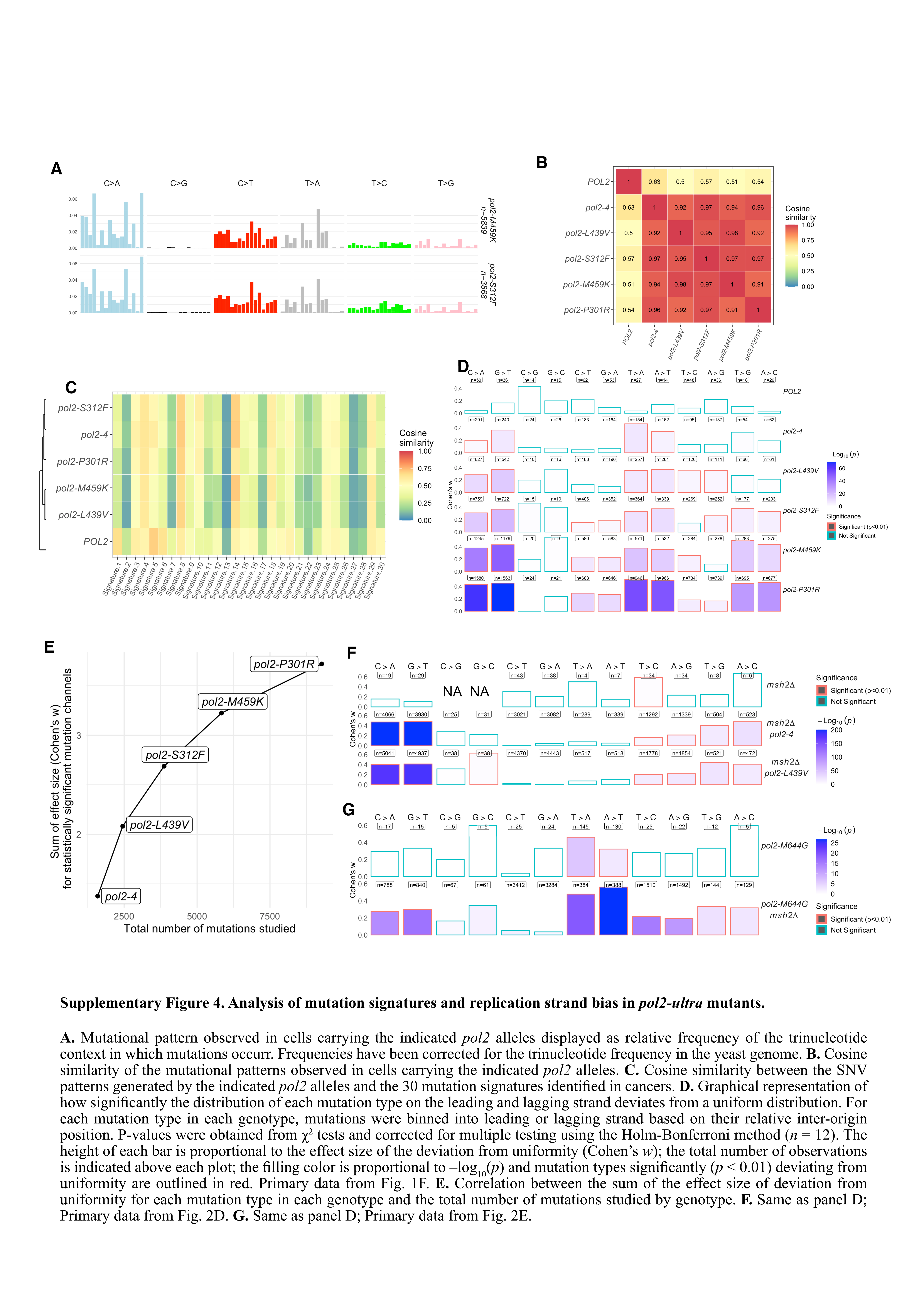

Supplement: gkab160_Supplemental_Files [file gkab160_supplemental_files.zip › SuppFig4.png]

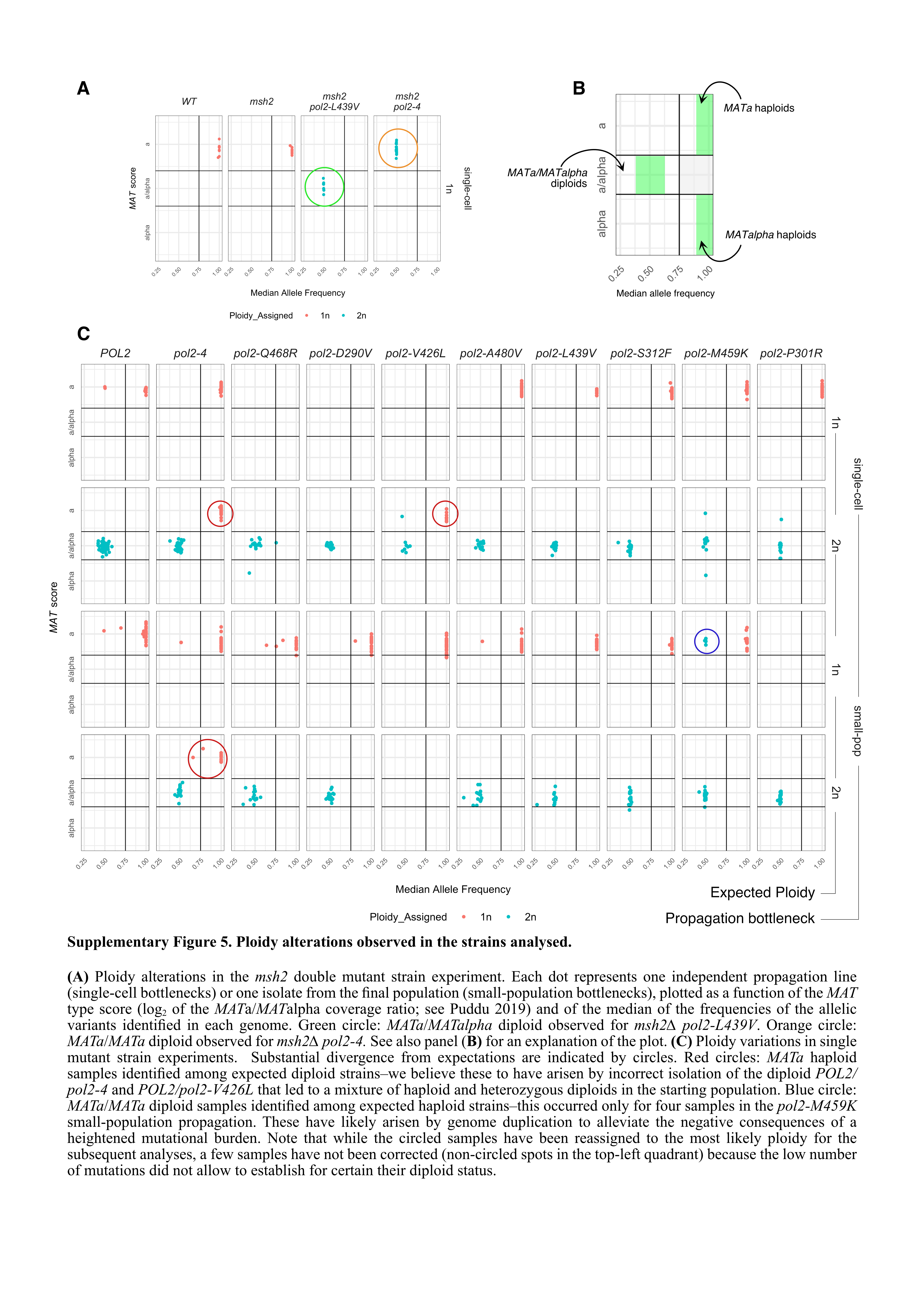

Supplement: gkab160_Supplemental_Files [file gkab160_supplemental_files.zip › SuppFig5.png]

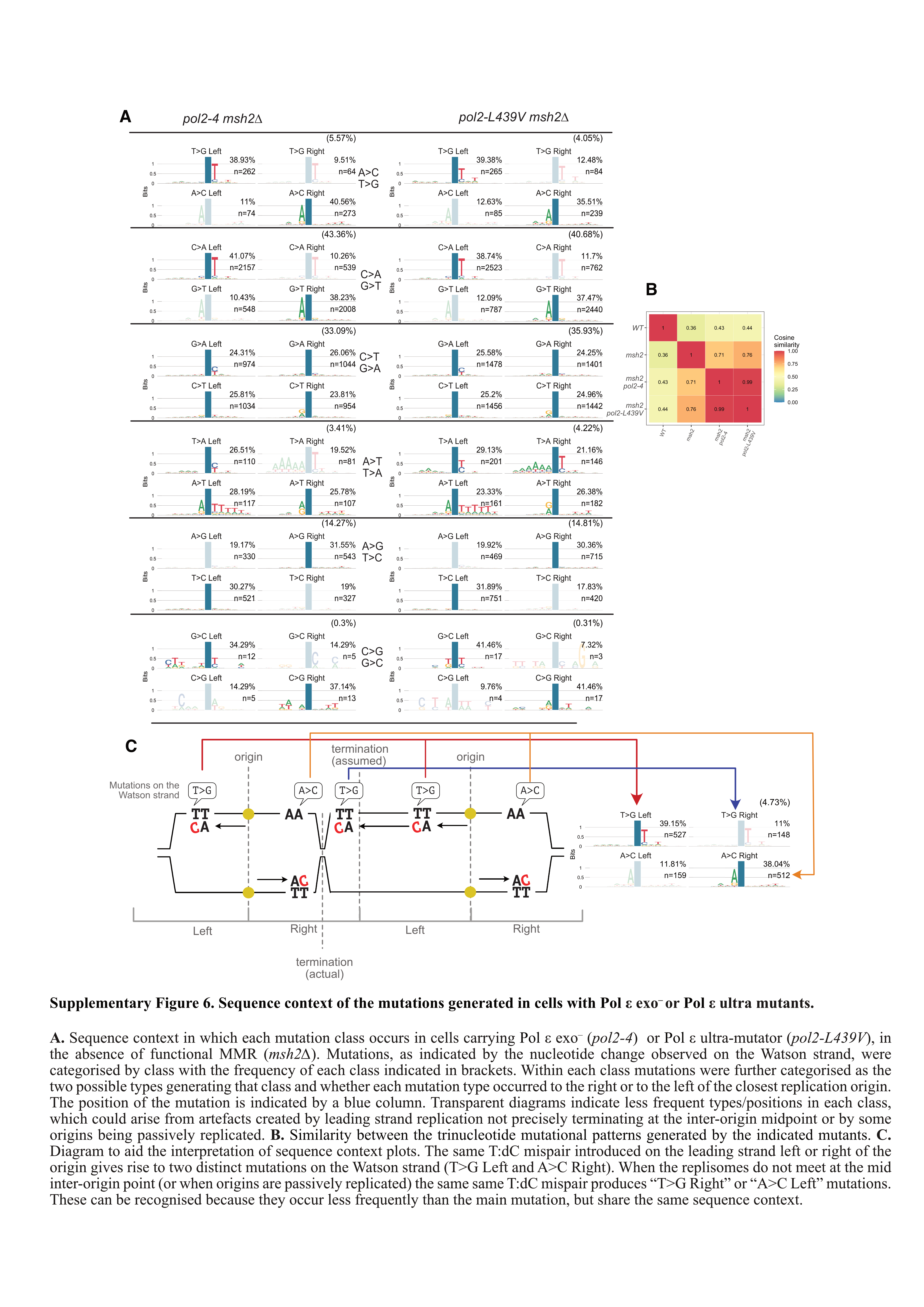

Supplement: gkab160_Supplemental_Files [file gkab160_supplemental_files.zip › SuppFig6.png]

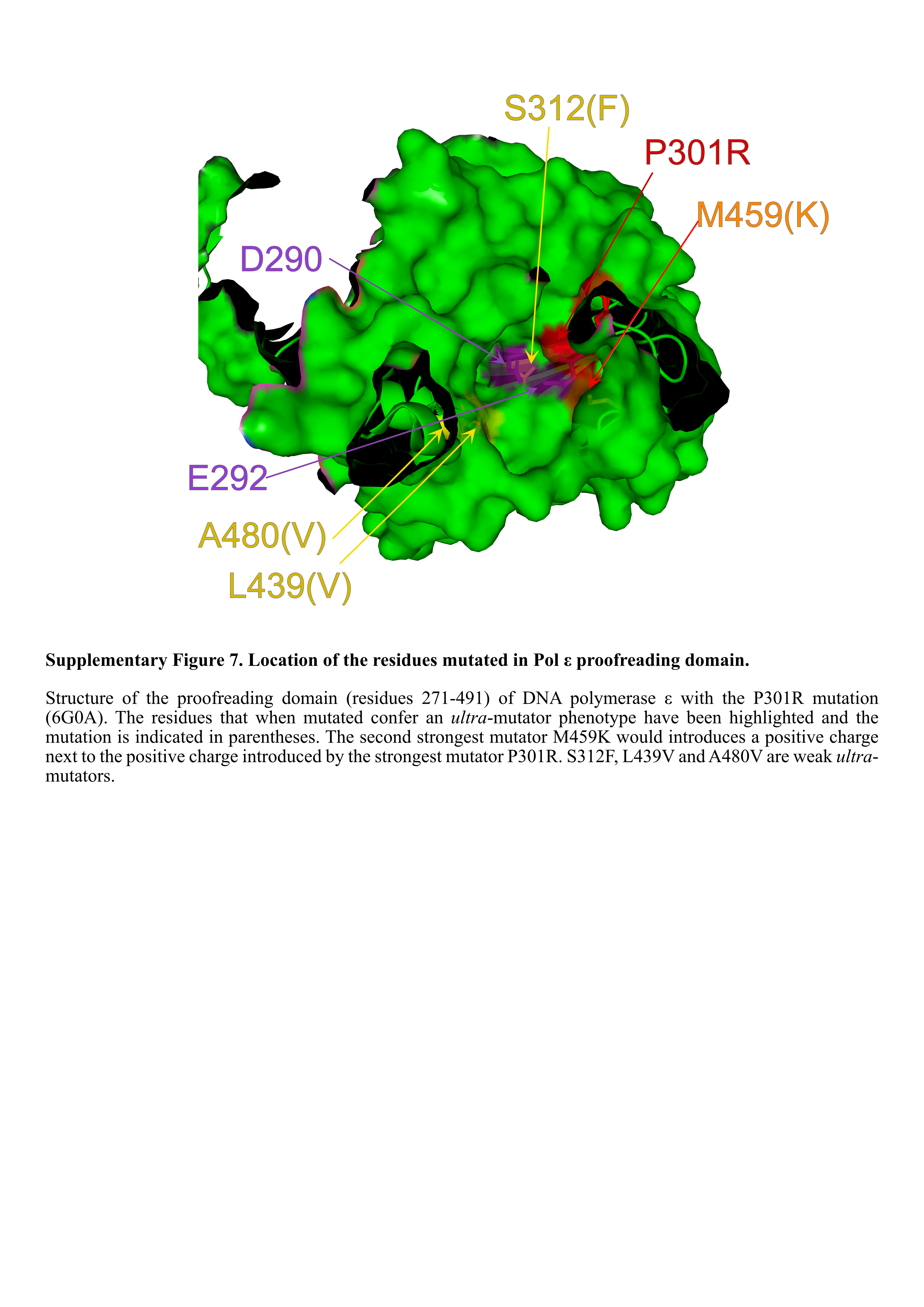

Supplement: gkab160_Supplemental_Files [file gkab160_supplemental_files.zip › SuppFig7.png]
